# Complex delayed blood transfusion reaction: A case report

**DOI:** 10.1097/MD.0000000000038467

**Published:** 2024-06-21

**Authors:** Qiang Li, Jinhui Xie, Jiali Sun, Kuo Fang, Tongtong Li

**Affiliations:** a State Key Laboratory of Experimental Hematology, National Clinical Research Center for Blood Diseases, Haihe Laboratory of Cell Ecosystem, Tianjin Key Laboratory of Cell Therapy for Blood Diseases, Institute of Hematology & Blood Diseases Hospital, Chinese Academy of Medical Sciences & Peking Union Medical College, Tianjin, China; b Tianjin Blood Center, Tianjin, China.

**Keywords:** allogeneic antibodies, case report, crossmatch, delayed hemolytic transfusion reaction (DHTR)

## Abstract

**Introduction:**

Inefficient blood transfusions present a significant challenge, leading to the wastage of crucial blood resources and increased medical expenses. This study aims to address this issue by providing a comprehensive analysis of a case involving an ineffective clinical transfusion and outlining the significance of identifying multiple alloantibodies in resolving transfusion challenges.

**Case report:**

We present a detailed follow-up on a patient treatment journey, highlighting the critical role of identifying multiple alloantibodies through various methodologies in addressing the transfusion problem. Subsequently, a strategic intervention was implemented, leading to a successful patient outcome.

**Conclusion:**

This study underscores the importance of conducting a thorough analysis of ineffective transfusions and implementing scientifically formulated transfusion plans based on rational explanations. Such an approach not only improves hemoglobin levels but also contributes to better patient outcomes, thereby reducing blood resource wastage and medical costs.

## 1. Introduction

Inefficient blood transfusions, encompassing both acute and delayed hemolytic transfusion reactions (DHTRs), represent a significant challenge in contemporary healthcare settings. Acute reactions manifest with immediate symptoms such as headache, lumbago, chills, fever, nausea, vomiting, and low blood pressure, even with small-volume transfusions. Conversely, delayed reactions are subtler, extending over a prolonged period, and are easily overlooked. These reactions demand heightened clinical attention due to their potentially severe consequences.^[1]^

These transfusion reactions occur due to various factors, including alloantibodies in the recipient serum that react with antigens present on transfused red blood cells. In cases of delayed reactions, the symptoms may not be immediately apparent, leading to multiple transfusions within a short timeframe without proper investigation.^[2]^

The prevalence of inefficient blood transfusions poses a significant burden on healthcare systems worldwide. It results in the wastage of precious blood resources, elevated medical costs, and delays in patient treatment. Addressing this issue is crucial not only for optimizing patient care but also for ensuring the efficient utilization of healthcare resources. In this report, we present a detailed follow-up on a case involving an ineffective clinical transfusion. We highlight the importance of vigilant analysis and intervention in addressing transfusion-related challenges. By elucidating the underlying causes and implementing strategic interventions, we aim to mitigate the risks associated with inefficient blood transfusions and improve patient outcomes. This report encompasses a comprehensive overview of the case, including the identification of alloantibodies, the methods employed for diagnosis, and the subsequent management strategies implemented to achieve a successful outcome for the patient.

## 2. Case report

The patient, a 54-year-old female with a 3-month history of myelodysplastic syndrome, presented with symptoms of fatigue, weakness, and shortness of breath consistent with anemia.

She had undergone multiple transfusions due to her condition. Before the second transfusion, anti-M antibodies were identified in her serum, prompting the administration of M antigen-negative red blood cells with confirmed compatibility in both major and minor crossmatches. Continuous transfusion of M antigen-negative blood was maintained until the detection of a minor non-crossmatch issue. Despite no additional irregular antibodies being identified through conventional matching tests, subsequent transfusions proved ineffective, showing no clinical improvement.

Ultimately, through various methods, including microcolumn gel, direct centrifugation, and papain 2-step methods, irregular antibodies including anti-E and anti-Jka were identified, resolving the transfusion issue. The patient blood transfusion successfully achieved the goal of alleviating symptoms related to anemia.

Detailed timeline of interventions and patient condition changes:

June 29: Anti-M antibodies identified.

July 11: Anti-Jka antibodies evident.

July 16: Anti-E antibodies detected.

July 21: Enhanced testing method utilized, confirming anti-E antibodies.

July 25: All 3 antibodies (anti-M, anti-Jka, and anti-E) conclusively identified.

July 25: Effective transfusions resumed after the administration of corresponding antigen-negative blood transfusion.

### 2.1. Criteria for determining transfusion effectiveness

The effectiveness of transfusions was evaluated based on clinical improvement in the patient symptoms of anemia, including fatigue, weakness, and shortness of breath.

### 2.2. Patient background

The patient, a 54-year-old female, had a medical history significant for myelodysplastic syndrome diagnosed 6 months prior to presentation, characterized by cytopenias and dysplastic changes in bone marrow morphology. She had previously received multiple transfusions due to symptomatic anemia secondary to her underlying condition. Additionally, she had a history of hypertension controlled with medication and a remote history of smoking cessation ten years ago. No other relevant medical conditions or significant family history were noted.

## 3. Materials and methods

### 3.1. Reagents

The Rh system antisera used in the study comprised anti-D antibody (from Shanghai Blood Biomedical Corporation), anti-E antibody, anti-C antibody, anti-e antibody, and anti-c antibody (all obtained from CE-Immundiagnostika). For the MNS blood group, antisera included anti-M and anti-N (both from CE-Immundiagnostika). In the Kidd system, antisera comprised anti-Jka (from CE-Immundiagnostika) and anti-Jkb (from Sanquin). Additional reagents employed in the study encompassed a Screening panel (from Sanquin), ID-Cards LISS/Coombs (from BioRad, Hercules, CA, USA), Makropanel (from Sanquin), and Acid Discharge Solution (from Sanquin).

## 4. Methods

The determination of irregular antibody specificity was carried out using various methods, each meticulously described for clarity and reproducibility. These methods included:

1. The microcolumn gel method: This method involved the use of gel cards for antibody detection and identification.
2. The 4 °C saline medium direct centrifugation method: Antibody specificity was assessed under specific conditions using centrifugation at 4 °C in a saline medium.
3. The papain 2-step method utilizing gel cards: Papain treatment was employed to enhance antibody detection on gel cards, facilitating accurate identification.
4. Absorption and dispersion methods: These techniques were utilized to further confirm antibody specificity, ensuring comprehensive analysis.
5. The acid-dispersion method: This method, executed in strict accordance with reagent instructions, enabled the determination of antigen specificity.
6. Antiglobulin testing using antihuman globulin microcolumns: This method provided additional confirmation of antibody presence through antigen-antibody complex detection.

## 5. Results

The results of antibody specificity tests are summarized in Table 1. Anti-M, anti-Jka, and anti-E antibodies were identified on June 29, July 16, and July 25, respectively. To ascertain the specificity of these antibodies, patient serum underwent an uptake and release test utilizing ONNccDEEJka-b+ cells (as detailed in Table 2). Notably, anti-M antibodies were detected in serum after a 30-minute absorption at 37 °C with spectral cells. At 4 °C, anti-Jka antibodies were identified using enzyme-treated spectral cells in gel cards, and anti-E antibodies were observed in gel cards with enzyme-treated cells after multiple washes of absorbed red blood cells. NS types were determined using the acid-dispersion method, as outlined in Table 3.

**Table 1:** Patient-specific antibody identification results.

	Blood group system																						Date					
	Rh-Hr					Kell				Duffy		Kidd		Lewis		P1	MNS				Luther		June 29		July 16		July 25	
	C	D	E	c	e	K	k	Kp^a^	Kp^b^	Fy^a^	Fy^b^	Jk^a^	Jk^b^	Le^a^	Le^b^	P1	M	N	S	s	Lu^a^	Lu^b^	NS	IAT	NS	IAT	IAT	Enzymes
1	+	+	0	0	+	+	+	0	+	+	+	+	+	+	0	+	+	0	+	0	0	+	4+	2+	4+	2+	2+	2+
2	+	+	0	0	+	+	+	0	+	+	0	0	+	+	0	0	+	0	0	+	0	+	4+	2+	4+	2+	2+	0
3	0	+	+	+	0	0	+	0	+	0	+	+	0	+	0	0	0	+	0	+	0	+	0	0	0	w+	1+	3+
4	0	+	0	+	+	0	+	0	+	0	0	+	0	0	+	+	0	+	0	+	0	+	0	0	0	1+	2+	3+
5	+	0	0	0	+	0	+	0	+	0	+	+	0	0	+	+	0	+	0	+	0	+	0	0	0	1+	1+	2+
6	0	0	+	+	0	0	+	0	+	+	+	0	+	0	+	+	+	+	+	+	0	+	2+	0	2+	0	0	W+
7	0	0	0	+	+	+	0	0	+	+	+	+	0	0	+	+	+	+	+	+	0	+	2+	0	2+	w+	w+	3+
8	0	0	0	+	+	+	+	0	+	0	+	+	0	+	0	+	+	0	+	0	0	+	4+	1+	4+	2+	2+	2+
9	0	0	0	+	+	0	+	0	+	+	0	0	+	0	+	+	+	0	+	+	+	+	4+	2+	4+	2+	2+	0
10	0	0	0	+	+	0	+	0	+	+	0	+	0	+	0	+	+	0	0	+	0	+	4+	2+	4+	3+	3+	2+
11	+	+	+	0	+	0	+	0	+	+	0	+	+	0	+	0	+	+	+	+	+	+	2+	W+	2+	w+	w+	2+
12	w	+	+	+	0	0	+	0	+	+	0	0	+	0	+	+	+	+	+	+	0	+	2+	0	2+	0	0	w+
13	+	0	0	+	+	0	+	0	+	+	*+*	0	+	0	+	0	0	+	+	+	0	+	0	0	0	0	0	0
14	0	0	0	+	+	0	+	+	+	0	*+*	+	0	0	0	+	0	+	0	+	+	+	0	0	0	w+	2+	2+
15	0	+	+	+	0	0	+	0	+	+	+	+	0	+	0	+	+	+	+	+	0	+	2+	0	2+	1+	1+	2+
16	0	0	0	+	+	0	+	0	+	0	+	0	+	+	0	0	+	+	0	+	0	+	2+	0	2+	0	0	0
Self-control	–	–	–	–	–	–	–	–	–	–	–	–	–	–	–	–	–	–	–	–	–	–	0	0	0	w+	w+	1+

**Table 2:** Antibody identification results after uptake and release using ONNccDEEJka− b+ cells (July 25).

	Rh-Hr					Kell				Duffy		Kidd		Lewis		P1	MNS				Luther		Post-absorption serum		Dispersion solution enzymatic method
	C	D	E	c	e	K	k	Kp^a^	Kp^b^	Fy^a^	Fy^b^	Jk^a^	Jk^b^	Le^a^	Le^b^	P1	M	N	S	s	Lu^a^	Lu^b^	NS	Enzymes	
1	+	+	0	0	+	+	+	0	+	+	+	+	+	+	0	+	+	0	+	0	0	+	4+	2+	0
2	+	+	0	0	+	+	+	0	+	+	0	0	+	+	0	0	+	0	0	+	0	+	4+	0	0
3	0	+	+	+	0	0	+	0	+	0	+	+	0	+	0	0	0	+	0	+	0	+	0	3+	1+
4	0	+	0	+	+	0	+	0	+	0	0	+	0	0	+	+	0	+	0	+	0	+	0	2+	0
5	+	0	0	0	+	0	+	0	+	0	+	+	0	0	+	+	0	+	0	+	0	+	0	2+	0
6	0	0	+	+	0	0	+	0	+	+	+	0	+	0	+	+	+	+	+	+	0	+	2+	0	1+
7	0	0	0	+	+	+	0	0	+	+	+	+	0	0	+	+	+	+	+	+	0	+	2+	3+	0
8	0	0	0	+	+	+	+	0	+	0	+	+	0	+	0	+	+	0	+	0	0	+	4+	2+	0
9	0	0	0	+	+	0	+	0	+	+	0	0	+	0	+	+	+	0	+	+	+	+	4+	0	0
10	0	0	0	+	+	0	+	0	+	+	0	+	0	+	0	+	+	0	0	+	0	+	4+	2+	0
11	+	+	+	0	+	0	+	0	+	+	0	+	+	0	+	0	+	+	+	+	+	+	2+	2+	w+
12	w	+	+	+	0	0	+	0	+	+	0	0	+	0	+	+	+	+	+	+	0	+	2+	0	1+
13	+	0	0	+	+	0	+	0	+	+	*+*	0	+	0	+	0	0	+	+	+	0	+	0	0	0
14	0	0	0	+	+	0	+	+	+	0	*+*	+	0	0	0	+	0	+	0	+	+	+	0	2+	0
15	0	+	+	+	0	0	+	0	+	+	+	+	0	+	0	+	+	+	+	+	0	+	2+	2+	1+
16	0	0	0	+	+	0	+	0	+	0	+	0	+	+	0	0	+	+	0	+	0	+	2+	0	0
Self-control	–	–	–	–	–	–	–	–	–	–	–	–	–	–	–	–	–	–	–	–	–	–	0	1+	

**Table 3:** Retrospective survey of patient plasma antibodies.

Test date enzyme treatment of cells	July 1	July 3	July 6	July 9	July 11	July 16	July 18	July 21	July 25
CCeeMMJKa+ b−	0	0	0	0	w+	1+	1+	1+	2+
CcEENNJKa− b+	0	0	0	0	0	0	0	w+	w+
cceeMNJKa+ b+	0	0	0	0	0	w+	w+	w+	1+

Anti-Jka antibodies were already evident on July 11, and anti-E antibodies were detected on July 21 when patient serum underwent testing utilizing an enhanced method involving enzyme-treated cells added to gel cards (refer to Table 4).

**Table 4:** Follow-up survey of blood transfusion records (MN, RH, and Kidd antigens) and patient antibody test results.

Blood transfusion time	Related red blood cell antigens			Detection of antibodies	Cross-matching results		DAT	Blood transfusion species
	MN	RH	Kidd		Major crossmatch	Minor crossmatch		
20160626	MN	CcEe	Jka+ b+	Negative	Negative	Negative	Negative	Red blood cells
20160629	NN	CcEe	Jka+ b−	Anti-M	Negative	Negative	Negative	Red blood cells
20160701	NN	Ccee	Jka− b+	Anti-M	Negative	Negative	Negative	Red blood cells
20160703	NN	CCee	Jka+ b+	Anti-M	Negative	Negative	Negative	Red blood cells
20160706	NN	CCee	Jka+ b−	Anti-M	Negative		Positive	Red blood cells
20160709	NN	Ccee	Jka− b+	Anti-M	Negative	–	Positive	Red blood cells
20160711	NN	Ccee	Jka+ b+	Anti-M	Negative	–	Positive	Red blood cells
20160716	NN	CCee	Jka− b+	Anti-M, Anti-Jka	Negative	–	Positive	Red blood cells
20160718	NN	CcEE	Jka− b+	Anti-M, Anti-Jka	Negative	–	Positive	Red blood cells
20160721	NN	CcEe	Jka− b+	Anti-M, Anti-Jka	Negative	–	Positive	Red blood cells
20160725	NN	CCee	Jka−	Anti-M, Anti-Jka, Anti-E	Negative	–	Positive	No blood transfusion

**Figure 1.**
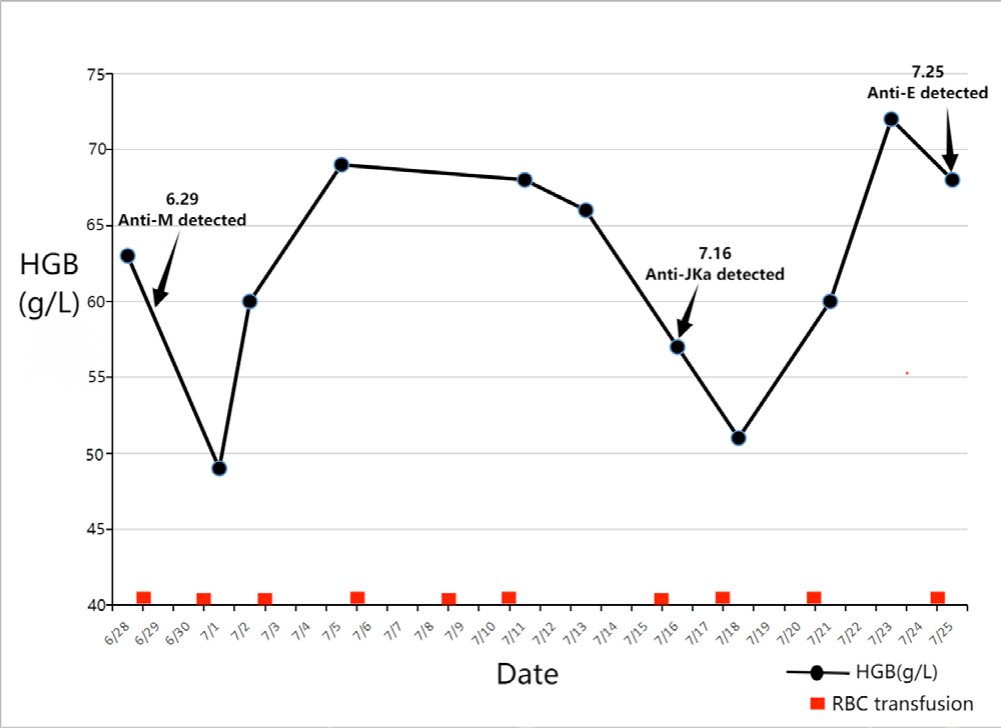
Patient transfusion results from June 26, 2016 to October 10, 2016 (d). The graph shows that red blood cells increased significantly when blood lacking corresponding antigens was transfused, while red blood cells decreased when blood with antigens corresponding to detected antibodies was transfused.

Following the identification of 3 antibodies (anti-M, anti-Jka, and anti-E) on July 25, 2016, there were no instances of further transfusion ineffectiveness after the administration of corresponding antigen-negative blood transfusion (as illustrated in Fig. 1).

## 6. Discussion

Undetected low-affinity antibodies, low-frequency antibodies, and dosage effects stand as major contributors to ineffective transfusions.^[3,4]^ Patients with a history of pregnancy and multiple transfusions face an elevated risk of ineffective transfusions, yet the detection rate remains low, potentially leading to acute and DHTRs.^[5,6]^ In this particular case, the patient received 3 transfusions of 12 units of M antigen-negative suspended red blood cells from June 29 to July 3. Both major and minor crossmatches yielded negative results, and the direct antibody tests of the patient were also negative. However, on July 6, the patient blood exhibited a weakly positive secondary response, and direct antibodies turned positive. This scenario should be a cause of significant concern for staff overseeing blood matching, as positive direct antibody results and secondary test matching are likely indicative of the emergence of new homologous antibodies. Several transfusions between July 6 and July 16 yielded unsatisfactory results. Anti-JKa antibodies were detected on July 16, and despite transfusing NNJka- blood, ineffectiveness persisted. Consequently, on July 25, a more sensitive method was employed to test for antibodies, revealing the presence of anti-E antibodies. Subsequently, the patient received an NNJka-ee blood transfusion, leading to a rebound in hematocrit and the alleviation of anemia symptoms.

Our findings underscore the importance of thorough analysis and reasoned decision-making when red blood cell transfusion proves ineffective. Rather than persisting blindly with transfusions in the pursuit of improving hemoglobin levels, it is crucial to identify and understand the underlying reasons, and formulate a scientifically sound transfusion plan.

The clinical impact of component transfusion can be categorized into 3 levels: an absence of clinical transfusion reactions; effective supplementation of specific blood components; and adjunctive treatment of diseases involving the regulation of physiological immune responses, among other factors. In the context of red blood cell transfusion, heightened attention should be directed not only toward hemoglobin elevation but also to immune responses and their physiological effects on red blood cells.

Our study also sheds light on cases involving ccDEe or ccDEE phenotypes, where the transfusion of 2 to 14 units of C-positive blood reportedly led to hemolysis 5 to 9 days later in 5 patients. The escalation of antibody responses correlates with an accelerated destruction of red blood cells. This type of transfusion response unfolds gradually, without significant activation of the coagulation system or the release of large amounts of vasoactive substances. While patients are often asymptomatic or exhibit mild clinical manifestations, this DHTR can insidiously harm the patient and potentially lead to side effects.^[5]^

The absence of clotting in saline and enzymatic blood before the initial transfusion post-admission suggests the absence of detectable anti-E antibodies in our patient at that time. Consequently, we infer that the presence of anti-E antibodies in the serum 15 days later was triggered by the E antigen during the initial transfusion. This delayed hemolytic reaction, attributed to immune Rh antibodies, primarily involves extravascular hemolysis, manifesting predominantly as xanthogranuloma and anemia, with hemoglobinuria often being inconspicuous or absent.^[6]^ In our case, the antiglobulin test was negative before transfusion, but high-affinity anti-E antibodies were detected 15 days after the transfusion of E antigen-containing blood. Positive results in direct antiglobulin and absorption release tests, along with increased total bilirubin and xanthogranuloma, were observed. However, hemoglobin levels did not increase, aligning with a diagnosis of DHTR. This reaction typically occurs 1 to 2 weeks posttransfusion, with symptoms primarily manifesting as xanthogranuloma, hemoglobinuria, and lumbar pain, which may be subtle and easily overshadowed by the underlying disease. Hence, in cases of unexplained xanthogranuloma and hemoglobin decrease posttransfusion, exploring the possibility of a delayed hemolytic reaction is crucial. Tracing blood transfused 1 to 2 days earlier and within the prior 1 to 2 weeks aids in determining the cause and facilitating appropriate treatment measures.

Normally, a recipient hemoglobin increases by approximately 10 g/L following the transfusion of 2 units of red blood cells, warranting the attention of the overseeing physician if the result significantly deviates from the expected value with an increasing trend. Bilirubin emerges as a sensitive indicator of hemolytic disease, initiating a rise within 1 to 2 hours of acute hemolysis and peaking at 5 to 7 hours. However, it may dissipate after 24 hours, coinciding with the restoration of liver and excretion functions to normal.^[1]^ Our patient exhibited abnormal liver and renal function, which might have been reflected in several biochemical parameters if monitored promptly. Continuous mild fever should be treated seriously by the physician.^[7]^ In such cases, re-transfusion should be halted, the cause identified, and effective transfusion administered promptly.

The data analysis of this case report has certain limitations. First, the sample size may be limited, which could affect the generalizability of the findings. With a small sample size, drawing broader conclusions about the effectiveness of the transfusion strategy proposed in the case becomes challenging. Second, there might be selection bias inherent in patient selection or data collection methods, potentially introducing result biases. For instance, the patient population included in the case report may not represent all those undergoing similar transfusion therapies. Furthermore, variations in assessment timing among patients could affect the consistency and accuracy of data analysis. Variability in laboratory testing, clinical assessments, and transfusion interventions can introduce confounding factors into the analysis. Additionally, there are limitations in the methods used to identify and interpret antibody specificity, potentially leading to differences in results or misunderstandings. Factors such as antibody titers, cross-reactivity, and assay sensitivity may affect the accuracy of antibody identification and characterization. Lastly, the duration of follow-up may vary, which could impact the observed outcomes. Long-term follow-up is crucial for assessing the sustainability of transfusion interventions and their impact on patient prognosis.

While complete prevention of DHTR may be challenging, efforts to minimize its occurrence can be made by providing Rh-compatible blood matched for clinically significant types in the patient. Concurrently, enhanced detection of C/c, E/e, K, Fya/Fyb, Jka/Jkb, M/N, and S/s is recommended to minimize harm caused by DHTR.^[8,9]^

## Author contributions

**Conceptualization:** Qiang Li, Jinhui Xie, Jiali Sun, Kuo Fang, Tongtong Li.

**Data curation:** Qiang Li, Jinhui Xie, Jiali Sun, Kuo Fang, Tongtong Li.

**Writing – original draft:** Qiang Li, Jinhui Xie, Jiali Sun, Kuo Fang, Tongtong Li.

**Writing – review & editing:** Qiang Li, Jinhui Xie, Kuo Fang, Tongtong Li.

**Formal analysis:** Jinhui Xie, Kuo Fang, Tongtong Li.

**Visualization:** Jiali Sun.
